# Treatment for Severe Malaria: Post-Artesunate Delayed Haemolysis and Neutropenia

**DOI:** 10.3390/healthcare10030413

**Published:** 2022-02-22

**Authors:** Mariangela Martino, Cecilia Liberati, Benedetta Bua, Elisa Barbieri, Paola Costenaro, Costanza Di Chiara, Carlo Giaquinto, Ettore De Canale, Osvalda Rampon, Daniele Donà

**Affiliations:** 1 Division of Paediatric Infectious Diseases, Department for Woman and Child Health, University of Padua, 35128 Padua, Italy; mariangela.martino@aopd.veneto.it (M.M.); elisa.barbieri@unipd.it (E.B.); paola.costenaro@phd.unipd.it (P.C.); costanza.dichiara@phd.unipd.it (C.D.C.); carlo.giaquinto@unipd.it (C.G.); osvalda.rampon@aopd.veneto.it (O.R.); daniele.dona@unipd.it (D.D.); 2 Division of Neonatal Intensive Care Unit, Department for Woman and Child Health, University of Padua, 35128 Padua, Italy; benedetta.bua@aopd.veneto.it; 3 Microbiology and Virology Unit, Department of Integrated Diagnostic Service, University of Padua, 35128 Padua, Italy; ettore.decanale@aopd.veneto.it; 4 Paediatric Infectious Disease Research Group, St. George’s University of London, London SW17 0QS, UK

**Keywords:** artesunate, post-artesunate delayed haemolysis, neutropenia

## Abstract

Parenteral artesunate (AS) is the WHO first-line treatment recommended in adults and children for severe malaria. Post-artesunate delayed haemolysis (PADH) is an uncommon adverse reaction to AS with a mechanism that is not fully understood, occurring in adults and children. Neutropenia is another possible finding after AS treatment, albeit rare. We present the case of a child who experienced both effects after treatment with AS for imported severe Falciparum malaria with very high parasitaemia. In addition, thirty-five paediatric cases of PADH, five cases of delayed anaemia without known haemolysis, and fourteen cases of neutropenia after artesunate treatment were identified from the literature review. PADH seems to be a dose-independent reaction and is not strongly related to hyperparasitaemia, although it is more frequent in this case. To date, the benefits of AS outweigh its potential side effects. However, haematological follow-up is mandatory to avoid possible complications from anaemia and neutropenia, especially in children treated with other contemporary drugs.

## 1. Introduction

Malaria infection creates a medical urgency in the paediatric population as progression can be rapid, especially in younger children, and it carries the most significant mortality risk in the first 24 h after presentation [1]. Most severe malaria cases worldwide are caused by *P. falciparum*, and clinical manifestations are related to end organ dysfunction. Therefore, patients with a *P. falciparum* parasitaemia > 10% are considered to have severe malaria even if they do not have organ involvement [2,3]. Prompt therapy is the key for stopping the progression of the disease. Parenteral artesunate (AS) is the first-line treatment recommended in adults and children with severe malaria, showing an increased survival rate compared to quinine and an excellent safety profile [2,3]. However, uncommon haematological adverse reactions are described, and data on paediatric patients are limited. Namely, post-artemisinin delayed haemolysis (PADH) in adults has been observed 1–3 weeks after initiation of treatment with artemisinin-based antimalarials such as artesunate, particularly in patients returning from endemic regions and presenting with hyperparasitaemia [4]. PADH is probably due to en masse haemolysis of damaged erythrocytes a short time after the removal of nonvital parasites killed by artesunate (releasing afterwards the “pitted” or “once infected” erythrocytes) by the spleen, which can be significant in the case of hyperparasitaemia. Therefore, monitoring the blood cell count at 7, 14, and 21 days after treatment is recommended [3]. Neutropenia after artesunate has also been reported, but data are fewer and limited mainly to adults. Furthermore, pathophysiology and clinical significance are widely not understood.

We present the case of a child who experienced both effects after treatment for imported severe Plasmodium falciparum malaria with very high parasitaemia, and review the available evidence on post-artesunate heamolysis and neutropenia.

## 2. Case Presentation

**Figure 1:**
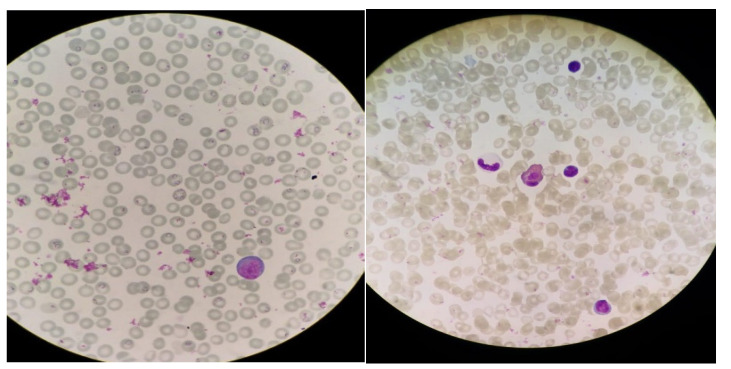
Light microscopy images of thin blood smears. Plasmodium falciparum trophozoites infecting red blood cells at the first day of hospitalization, before artesunate treatment (**left**). Parasitaemia clearance after second dose (**right**).

A 15-month-old child presented to our Paediatric Hospital in Padua, Italy with a high fever five days after a recent permanence in Nigeria. Antimalarial prophylaxis was never administered to the child. His mother reported that he was treated for malaria (with unknown intramuscular medicines for three days) two months before. At the admission to the emergency department, the child appeared well and active without any neurological signs, and physical examination revealed only mild spleen and liver enlargement. Blood tests showed thrombocytopenia (16,000/mmc) and moderate normocytic anaemia (Hb 9.5 g/dL). Severe malaria was diagnosed through the peripheral blood smear, revealing *P. falciparum* with hyperparasitaemia (15% red blood cells parasitized) (Figure 1). Intravenous therapy with 3 mg/kg of artesunate was immediately started: three doses were administered every 12 h, and afterwards, the same dose was repeated up to five total administrations (0–12–24–48–72 h). Monitoring of parasitaemia showed a rapid drop to 1–3% after the first dose and to clear after the second. However, despite the favourable response, therapy was continued parenterally because of the poor compliance to oral capsules. The fever resolved in 48 h, with the recovery of platelets and stable haemoglobin levels (9.2 mg/dL) on day four. The patient was discharged without antimalarial therapy. 

During follow-up visits, the patient remained asymptomatic but presented on day seven a haemoglobin count decrease, up to 7.7 mg/dL, with 12.10% reticulocytes, MCV 71.7 fl, and haptoglobin <0.08 g/L, compatible with haemolytic anaemia. On day 14, haemoglobin was stable (7.9 mg/dL), but severe neutropenia (230/mmc) was noted, and on day 21, gradual resolution of anaemia (9.2 mg/dL) and normal neutrophils count (4110/mmc) were observed.

## 3. Materials and Methods

We searched all full-text, peer-reviewed publications and online databases about post-artesunate delayed haemolysis and neutropenia. 

English language articles were searched using PubMed, Google Scholar, Embase, and Cochrane through November 2021. The search terms utilised were “malaria”, “artesunate”, “anaemia”, ”neutropenia”, and “children”. The identified articles were screened for inclusion independently by two authors. The eligibility criterion for inclusion was a description of post-artesunate delayed haemolysis or anaemia and/or neutropenia in paediatric patients (<18 years of age). PADH was defined as anaemia following artesunate treatment with evidence of a haemolytic process (low haptoglobin and increased lactic acid dehydrogenase, bilirubin, and reticulocytes). Broader reports of delayed anaemia occurring >7 days after artesunate without known evidence of haemolysis were also included, as definitions among studies were very different. Demographic data, laboratory and microbiological tests, treatments, and outcomes were retrieved. Hyperparasitaemia was considered >4% [2]. 

Moreover, online databases screened for post-artesunate delayed haemolysis and neutropenia were VigiAccess [5] (WHO Collaborating Centre for International Drug Monitoring) and EudraVigilance [6], the European database of suspected adverse drug reaction reports (EMA). We conducted a search using VigiAccess on the VigiBase^®^ database on the 30th of November to search for blood adverse drug reactions (ADRs) related to artesunate. The EudraVigilance database webpage tool was accessed on 30th of November and the term “Artesunate” was searched in the query by substance.

Categorical data were summarized as frequency counts and percentages. Continuous data were summarized using the mean and the interval range.

## 4. Results of Literature Review

The literature review identified thirty-five eligible paediatric cases of delayed haemolysis after artesunate treatment for malaria and five cases of delayed anaemia without known haemolysis. Data for PADH are summarized in Table 1.

### 4.1. Definition of PADH and Incidence in the Paediatric Population

The first evidence of anaemia with signs of haemolysis after the resolution of acute infection and parasite clearance in patients treated with artesunate was collected in 2010 from adult patients. In 2014, Burri et al. described five paediatric cases of delayed anaemia after artesunate treatment for severe malaria; data about possible mechanisms and haemolysis at that time were not evaluated [9]. Similarly, in an RCT comparing different AS schedules for severe malaria, anaemia at day 7 was found in 185 patients out of 972, with seven children remaining anaemic after day 7 [10]; a subgroup was actively monitored for signs of haemolysis in a substudy, resulting in five cases of PADH [8]. Afterwards, following evidence on adults and first reports on children, delayed haemolysis after artesunate treatment was established as an autonomous entity for its presumed pathophysiology and course. PADH was better defined as a nonrecurring episode, with a 10% decrement in haemoglobin associated with haptoglobin < 0.1 g/L and an increase in LDH to > 390 U/L or a 10% rise > 7 days after treatment initiation with artesunate [18,19]. However, definitions of PADH and laboratory parameters evaluated are highly heterogeneous in the reported paediatric cases, and many variables influenced the case definitions, such as the patient’s background (native or traveller) and location of care (endemic or nonendemic region), making it challenging to operate a reliable incidence estimation and comparison. 

A systematic review in 2014 described an overall rate of delayed haemolysis of 13% after usage of artemisinin derivatives, reporting cases in 31 adults and six children [20]. Five children were treated for severe malaria in endemic regions [8], while one was a returning traveller treated in Europe [7]. In the same year, a report from Congo described delayed haemolysis in five native children younger than five years treated with AS [9], and Scheu in 2019 added two additional cases from the same region [12]. In 2020, a prospective case series of 91 Ugandan children with severe malaria treated with IA showed no cases of PADH [21]. A prospective collection of 1391 patients treated for imported malaria in a nonendemic region (France) from 2011 to 2017 included 128 children, with 7 cases of PADH (5.4%) [17]. Therefore, the incidence of PADH in children treated with parenteral AS for severe malaria in these literature studies varied from 0 to 11%.

In our series, PADH was defined as anaemia after day 7 associated with laboratory signs consistent with haemolysis (increased LDH or reticulocytes and reduced haptoglobin).

In a case report by Taneja, a 6-month-old infant developed haemolytic anemia ten days after artesunate treatment for p.vivax infection, associated with major blood incompatibilities. A diagnosis of malaria-induced autoimmune haemolytic anemia was considered, but PADH could not be excluded [16].

For all patients, haemolysis and worsening of anaemia were described after parasite clearance, with a mean haemoglobin nadir of 6.4 g/dL (range: 1–10 g/dL).

### 4.2. Demographic and Malaria Characteristics of PADH Cases

Age was available for 19 patients, with a mean of 1.3 years (and a range of 6 months to 8 years). Sex was described in 15 cases, with equal distribution.

All patients contracted malaria in endemic regions (Africa and India), but not all cases were treated locally; 11 patients from four studies were returning travellers (VFR) treated in nonendemic countries.

Data about the parasitaemic count were available for 17 patients; five who developed PADH were not hyperparasitaemic at presentation, according to the WHO definition of hyperparasitaemia. Four of them acquired malaria in India, one was a child treated in Africa, and one was a returning traveller from Africa. 

The parasitaemia was unavailable for ten patients, but all of them had a parasitaemic count > 2% as an inclusion criterion [11]. The highest parasitaemia was 24% in a one-year-old child that went to Africa to visit friends or relatives [13]. 

Most of the children received standard doses of AS (2.4 mg/kg repeated up to five doses); five children received 4 mg/kg for three doses, and three children from India received 14–15 mg/kg. For 15 patients, treatment was completed with artemether-lumefantrine or artesunate-amodiaquine.

### 4.3. Treatment and Outcomes of PADH

Nine cases were treated with blood transfusions. Recovery was observed in all patients, with favourable outcomes.

### 4.4. Neutropenia

Neutropenia in children receiving artesunate was described only in a retrospective observational study conducted in India [22]. 

Fourteen out of thirty-one HIV-infected patients treated with artesunate plus amodiaquine for uncomplicated malaria developed neutropenia on day 14 after treatment; the risk was increased with contemporary antiretroviral therapy. In the same study, 15 out of 253 HIV-uninfected patients (6%) developed neutropenia within 14 days after initiation of artesunate plus amodiaquine. Overall, the median neutrophil count was 560 N/mm^3^, ranging from 400 to 680 [22].

### 4.5. Online Databases for Suspected Adverse Drug Reactions (ADRs)

A total of 2929 ADRs were retrieved using the term “Artesunate”. Among those cases, 227 were classified as blood and lymphatic system disorders: 137 were cases of “heamolytic anemia”, 28 of “haemolysis”, 10 of “thrombocytopenia”, 6 of “neutropenia”, 6 of “leukopenya”, 5 of “autoimmune haemolytic anaemia”, 4 of “Coombs positive haemolytic anaemia”, 3 of “agranulocytosis”, 2 of “intravascular haemolysis”, 1 of “aplastic anaemia”, 1 of “normochromic normocytic anaemia”, 1 of “granulocytopenia”, 1 of “lymphopenia”, and 1 of “pancytopenia”. Overall, 1657 out if 2929 ADRs were reported prior to the year 2000, and 2232 (76%) came from Asia. In 57% of cases (1672/2929), ADRs were reported in patients aged 18–44 years. Unfortunately, a limitation of VigiAccess is the impossibility for a stratification by age and type of ADRs.

To bypass this limitation, we accessed the EudraVigilance database via webpage and searched for “Artesunate” in the query by substance. The search retrieved no ADRs about artesunate.

## 5. Discussion

This paper reports a 15-month-old child affected by imported severe malaria with a very rapid reduction of the parasitaemia after a single dose of artesunate. However, following the treatment, anaemia and severe neutropenia were observed.

Artesunate (AS) is a semisynthetic derivative of artemisinin, the active antimalarial component of the herb *Artemisia annua*, which targets ring-stage parasites, preventing their maturation and inhibiting erythrocyte sequestration. Artesunate is water-soluble and is faster acting than other compounds of the same family. In 2010, the World Health Organization (WHO) changed the recommendation for first-line therapy for severe malaria in children and adults from intravenous quinine to intravenous artesunate. Large randomized trials have demonstrated that artesunate induces a more rapid parasite clearance, decreasing mortality in patients with complicated malaria [23,24].

The safety profile of parenteral artesunate in children trials was excellent, with no drug-related severe adverse effects reported [10,23,25]. However, these studies focused on immediate side effects, while delayed reactions were not addressed by most.

The assumed mechanism for PADH is the massive and synchronous destruction of the large proportion of once-infected erythrocytes (oi-RBC) in hyperparasitised children (but not only), after 7–15 days from the spleen removal of parasites killed by artesunate. The reason pitted cells are initially spared from haemolysis and then destroyed after about two weeks is unknown, but after the exhaustion of pitted erythrocytes, haemolysis does not recur. Interestingly, the proportion of pitted erythrocytes is significantly higher after using artemisinins than after quinine, an effect that may explain why delayed haemolysis is strongly associated with artesunate treatment [26].

In 2017, Fanello et al. illustrated the delayed haemolysis occurrence in African children with hyperparasitaemic *falciparum* malaria, randomized to parenteral quinine or artesunate. Overall, delayed haemolysis occurred in less than 10% of patients following artesunate (10 children), with a similar incidence rate in children treated with quinine [11]. However, the proportion of oi-RBC was three times higher in children receiving artesunate. In this study, the percentage of oi-RBC was analysed and compared to previous research on adult patients who were returning travellers [19]. In native children, the ratio of oi-RBC to initial parasitaemia was lower than returners, as was the percentage of them persisting at seven days. In conclusion, visitors returning to nonendemic regions have significantly higher oi-RBC. These findings suggest other possible mechanisms for removing infected cells in African children.

Furthermore, an Indian prospective cohort in 2020 identified 21 children treated with AS for severe malaria, among which 3 cases of delayed haemolysis were found. Notably, no children had baseline hyperparasitaemia, calling the role of the spleen removal of pitted cells into question [14]. Although at present there is no clear evidence for an immunologic component to haemolysis of oi-RBC, such as serum reaction or opsonization following injectable AS, this possibility cannot be ruled out. Presumably, people living in endemic regions have a malaria-immunity background which somehow influences parasitaemia and oi-RBC clearance, compared to naïve travellers. Other possible mechanisms of haemolysis require further investigation, especially in the paediatric population, e.g., genetic susceptibility, direct toxic effects of the drug, or drug metabolites with a longer half-life.

Autoimmune haemolytic anaemia is described in association with malaria infection in adult and paediatric populations; however, the role of an immune process underlying PADH has been questioned. Bartoli et al. analysed 39 cases of PADH (both adults and paediatrics cases) comparing clinical features, laboratory parameters, and outcomes of patients with and without a positive direct antiglobulin test (DAT). They found no differences and concluded that a DAT positivity may be a nonspecific expression of malaria-related systemic immune activation [27].

The influence of haemoglobinopathies and enzyme deficiencies on post-artemisinin derivatives haemolysis has not been investigated consistently; in the prospective study by Rolling, all five children who developed post-artesunate haemolysis had a hemoglobin AA genotype, while in the group without haemolysis four children (5%) had sickle cell trait (HbAS) and five children (8%) had haemoglobin C trait (HbAC) [8]. A direct toxic effect of artesunate or its metabolite dihydroartemisinin is also doubtful due to a half-life shorter than two hours [28].

To summarize, PADH seems to not be strictly related to baseline parasitaemia or a dose-dependent reaction. Indeed, it was observed in children treated with AS 5 mg/kg as total dose [13] and in children treated with 12 mg/kg total dose [8]. Our child was treated with five intravenous doses up to 15 mg/kg despite a parasitaemia clearance time of 24 h. It is not reasonable that this could cause PADH, but this addresses the lack of paediatric formulations in nonendemic regions. WHO guidelines highlight, indeed, the necessity to shift therapy to an oral combination regimen as soon as oral intake is possible but not before 24 h of parenteral treatment [2]. In nonendemic countries, paediatric formulations for artemisinin-based combination therapy like granules and oral suspensions are often not available, posing a challenge in treating smaller children and infants who may not be compliant with swallowing crushed tablets. A systematic review showed that paediatric formulations like dispersible tablets and suspensions in endemic regions are not associated with an increased treatment failure or adverse event rate. However, no study assessed the acceptability [29].

All paediatric cases presented recovered fully after PADH, though in more severe cases, blood transfusions were required. Thus, blood transfusions should be reserved for severe and symptomatic patients. Steroids are used in immune drug-induced haemolytic anemia for their immunomodulating effect, but their benefits remain undetermined. The role of steroids in PADH has not been evaluated, though some adults’ reports advocated the antibodies’ responsibility against artesunate and treated patients with steroids [30,31]. Steroids have been used in cases of autoimmune haemolytic anaemia (AIHA) associated with malaria infection, with a presumed immune mechanism detection of antibodies (direct Coombs test or blood incompatibilities) [32].

The impact of these findings should influence follow-up strategies after artesunate use, which could be extremely variable according to the setting of care. The monitoring of patients treated after returning from endemic regions to high-income countries is often easy to carry on, while it is challenging in a low-income endemic setting. Efforts are needed to stress the importance and accomplish the possibilities worldwide of haemoglobin monitoring up to 4 weeks after artesunate, as PADH is not rare in the paediatric population and can be severe.

Prognostic tools or scores to identify children at increased risk for artesunate side effects are lacking. Research is focusing on newer ways to ease diagnosis of malaria and prevent its severe evolution, especially in endemic areas, where basic infrastructures are often lacking. The implementation of such technology-driven or omics-based systems throughout endemic areas could prevent aggressive treatments and related side effects. [33,34,35,36,37].

### Neutropenia

The patient in the presented case experienced severe neutropenia on day 14 after treatment with artesunate. Neutropenia after artesunate has already been described. In 2010, a prospective study on adults was published, where high doses of oral artesunate were administered to 26 uncomplicated malaria patients in Cambodia to study if resistance to artesunate can be overcome by increased dosage. Interestingly, dose-dependent delayed neutropenia emerged from the analysis within 14 days after treatment, occurring in six patients (19%): adults taking 42 mg/kg total dose of artesunate significantly developed neutropenia compared to lower dosages, and this coincided with a slower plasma clearance of the drug [38]. However, other marrow cellular lines were not afflicted, excluding global myelotoxicity.

Neutropenia was observed in children treated with artesunate plus amodiaquine for uncomplicated malaria in Uganda, and incidence was significantly higher in HIV co-infected children undergoing contemporary antiretroviral therapy [22]. However, the data could be flawed as amodiaquine is known to cause neutropenia [39].

Several nonchemotherapeutic drugs have been shown to induce neutropenia or agranulocytosis. There are two assumed mechanisms for this: idiosyncratic reactions, which are dose-independent and are thought to be mediated by immunological removal of cells (increased neutrophil destruction), or suppressed bone marrow activity (decreased neutrophils production). For example, Quinine-induced neutropenia depends on antibodies’ formation reactive with neutrophils, and the exact mechanism motivates thrombocytopenia and lymphopenia [40]. A similar process could lead to neutropenia in patients treated with artesunate; indeed, temporal correlation is suggestive, as idiosyncratic-immune-mediated reactions usually occur 1–6 months after exposure or earlier after re-exposure. This is consistent with the time needed by T-cells to proliferate [41]. 

The child in our case presented with neutropenia at day 14 but not at day 7, while two patients from the Cambodian study had neutropenia as soon as three days after artesunate [38]. These times are shorter than what was expected with idiosyncratic reactions and what was observed with other neutropenia-induced drugs, such as beta-lactams [42]. However, re-exposition to the same agent can shorten the time for the immune-mediated mechanism to onset. All people from the Cambodian study were coming from an endemic area, where previous malaria treatments are possible, and our patient was treated two months before, as reported by his mother, and probably received artesunate intramuscular medication. 

Neutropenia resulting from immune-mediated reactions is usually self-limiting and does not increase the risk of infection as bone marrow has no damage, therefore, antibiotic prophylaxis is not warranted for brief neutropenia events. Thus, at last, neutropenia is frequently caused by infections themselves, especially in paediatrics and, most often, with viruses. This occurrence, however, has not been described in malaria.

The WHO VigiAcess database reported several cases of PADH and neutropenia after artesunate. However, a subanalysis according to age group was impossible with the tool. Therefore, healthcare professionals should implement reports of ADRs in the dedicated web systems to achieve a complete overview; especially in paediatrics, where literature reports are fewer and long to elaborate.

## 6. Conclusions

Post-artesunate delayed haemolysis can occur after seven days from parenteral artesunate treatment for severe malaria in the adult and paediatric population to a lesser extent, and returning travellers seem to be at an increased risk. Thus, awareness should be high in children as well. Although vigilance is warranted with follow-up Hb measurement, the benefits of artesunate appear to outweigh the risk of PADH. A there is no clear evidence that PAHD is a dose-dependent mechanism, attention should be paid to changing the therapy to oral artemisinin-combination as soon as the child tolerates oral intake. The lack of paediatric oral formulations, such as oral suspensions, should be addressed in nonendemic regions. 

Neutropenia is a possible immune-mediated reaction to artesunate and should be monitored, especially in the case of re-administration. No antibiotic prophylaxis is needed as it is usually self-limiting and does not increase the risk of severe infections. Caution should be greater in patients using other medications.

## Figures and Tables

**Table 1:** Previous cases of post artesunate delayed anaemia or haemolysis (PADH).

Reference	Study and Outcome	Definition of Delayed Haemolysis (or Anemia)	Cases	Age in y (Median, Interval)	Provenience	Plasmodium Species	Parasitemia (Median, Interval)	Antimalarial Treatment	Hb (g/dL) Nadir, Median (Interval)	Blood Transfusions, N (%)
Kreeftmeijer-Vegter et al. (2012) [7]	Retrospective case series (adults and children), safety and efficacy data of patients treated with IV artesunate	Defined by the course of haemoglobin, LDH and haptoglobin after day 7	1	5	VFR (Africa)	NA	12%	Q, AS 2.4 mg/kg for 3 doses, AA	3.8 at day 8	0
Rolling et al. (2014) [8]	Prospective study (children), incidence of post-artesunate delayed haemolysis on day 14	Any anaemia between day 7–14, any reduction in haptoglobin between day 7–14, LDH > 350 U/L at day 14	5	2.1	Africa	*P*. *falciparum*	6.2% (4–9%)	AS 4 mg/kg for 3 doses, AL	6.7 at day 14	1 (20%)
				(0.5–5)					(2.8–9)	
Burri at al. (2014) [9]	Prospective case series (children), incidence of post artesunate delayed anaemia during at least one follow-up visits	Anaemia: reduction of Hb level	5	1.7	Africa	*P*. *falciparum*	4.5% (n = 3.1 –8.7)	AS 2.4 mg/kg for 3 to 5 doses (1 patient), AA	4.36 (4.2–5) after day 8	4 (80%)
		Haemolysis was not evaluated		(0.5–3)						
Kremsner et al. (2016) [10]	RCT 3 doses AS IV or IM vs. 5 doses AS in African children. Reduction of parasitaemia, post-hoc analysis of delayed anaemia	Delayed anaemia: Hb < 7 g/dL after 7 days (in a post-hoc analysis).	185 at day 7.	NA	Africa	NA	NA	NA	NA	NA
		PADH was analysed in a subgroup (Rolling 2014)	2 after day 7.							
Fanello et al. (2017) [11]	RCT (IV artesunate or quinine) in hyperparasitaemic children, incidence of post-treatment haemolysis	Anaemia was defined as reduction ≥ 10% of Hb level between day 7–42 post-treatment; haemolysis was defined as increased LDH	10	NA	Africa	*P*. *falciparum*	>2%	AS 2.4 mg/kg for 4 doses	10.5 (4.7–12.5) at day 14	1 (10%)
Scheu et al. (2019) [12]	Retrospective case series (children), incidence of post-artesunate delayed haemolysis	Any anaemia between day 7–14, any reduction in haptoglobin between day 7–14, LDH > 350 U/L at day 14	2	NA	Africa	*P*. *falciparum*	NA	AS 2.4 mg/kg for at least 3 doses	10 at day 14	NA
Bélard at al. (2019) [13]	Retrospective case series (children) analysing efficacy and safety of IV artesunate	Defined by the course of haemoglobin, LDH and haptoglobin in children with follow up longer than 7 days	3	2.6	VFR (Africa)	*P*. *falciparum*	16% (9–24)	AS 2.4 mg/kg for 3 doses	7.5 after day 14	0
				(1–6)						
Savargaonkar et al. (2020) [14]	Prospective case series (children), incidence of post-artesunate delayed haemolysis	Defined by the course of Hb, LDH, reticulocyte count.	3	3.8 (2–7)	India	*P*. *falciparum*	<0.1%	AS 14–15 mg/kg in minimum 3 doses, AA	8.3 after day 7	1 (33%)
Patel et al. (2020) [15]	Case report of post-artesunate delayed haemolysis	Defined by the course of Hb, LDH, reticulocyte count.	1	8	VFR (Africa)	*P*. *falciparum*	1.5%	AS 2.4 mg/kg in minimum 4 doses then AL	1.0 at day 12	1
Taneja et al. (2019) [16]	Case report of autoimmune haemolytic anaemia after being treated with artesunate for P. vivax malaria	Defined by the course of Hb, LDH, reticulocyte count.	1	0.6	India	*P. vivax*	>2%	AS (dose NA)	2.8 at day 10	1
Roussell et al. (2021) [17]	Prospective Multicenter study (adults and children), clinical and epidemiologic features of people treated with intravenous artesunate	Defined by the course of haemoglobin, haematocrit, lactic acid dehydrogenase, bilirubin, and reticulocytes	7	Younger than 16	VFR (Africa)	NA	NA	NA	NA	NA

Q: quinine, AS: artesunate, AL: artemether-lumefantrine, AA: artesunate-amodiaquine, NA: not available, VFR: visiting friends and relatives, Hb: haemoglobin, LDH: lactic acid dehydrogenase.

## Data Availability

The data presented in this study are available on request from the corresponding author. The data are not publicly available due to privacy.
